# Immediate non-traumatic postmortem computed tomographic demonstration of myocardial intravascular gas of the left ventricle: effects from cardiopulmonary resuscitation

**DOI:** 10.1186/2193-1801-2-86

**Published:** 2013-03-07

**Authors:** Takahisa Okuda, Seiji Shiotani, Tomoya Kobayashi, Mototsugu Kohno, Hideyuki Hayakawa, Kazunori Kikuchi, Kunio Suwa

**Affiliations:** 1 Department of Legal Medicine, Nippon Medical School, Tokyo, Japan; 2 Department of Radiology, Tsukuba Medical Center, 1-3-1 Amakubo, Tsukuba, Ibaraki, 305-8558 Japan; 3 Department of Radiological Technology, Tsukuba Medical Center, Tsukuba, Japan; 4 Department of Critical Care and Emergency Medicine, Tsukuba Medical Center, Tsukuba, Japan; 5 Tsukuba Medical Examiner’s Office, Tsukuba, Japan; 6 Department of Pathology, Tsukuba Medical Center, Tsukuba, Japan; 7 Department of Biomedical Engineering, Teikyo Junior College, Tokyo, Japan

**Keywords:** Postmortem computed tomography (PMCT), Cardiopulmonary resuscitation (CPR), Cardiovascular gas (CVG), Supersaturation, Tribonucleation

## Abstract

An 87-year-old man was found in a state of cardiopulmonary arrest. Despite cardiopulmonary resuscitation (CPR) for over 1 hour by emergency technicians and physicians, the patient died. Immediate postmortem computed tomography showed cardiovascular gas in the right atrium, right ventricle, and left ventricle. Cardiovascular gas in the left ventricle was located in the myocardium and appeared as linear or branch-shaped suggesting the presence of myocardial intravascular gas. This is the first report describing the appearance and significance of myocardial intravascular gas of the left ventricle as a CPR-related change.

## Introduction

Due to the worldwide decline in conventional autopsy rates, the need for and frequency of postmortem imaging as a complementary, supplementary or alternative method for autopsy have increased worldwide (Brogdon 1998; Swift & Rutty 2006; Oesterhelweg & Thali 2009; Roberts et al. 2012; Wichmann et al. 2012; Takahashi et al. 2012; Okuda et al. 2012; Lundstrom et al. 2012; Daly et al. 2012). Interpreting whether findings are related to cause of death requires knowledge of normal postmortem computed tomography (PMCT) findings. PMCT findings are classified into three categories (Takahashi et al. 2011): (1) causes of death and associated changes, (2) postmortem changes, and (3) cardiopulmonary resuscitation (CPR)-related changes. Intravascular gas as CPR-related changes on early non-traumatic PMCT obtained within a few hours after confirmation of death have been reported including hepatic vascular gas, cerebral vascular gas, and cardiovascular gas (CVG) (Shiotani et al. 2004; Shiotani et al. 2005; Yokota et al. 2009; Takahashi et al. 2009; Shiotani et al. 2010; Zenda et al. 2011; Ishida et al. 2011). Laurent et al. verified that CPR induces intravascular gas using porcine experimental models (Laurent et al. 2013).

CVG on early non-traumatic PMCT often shows gas bubbles along the anterior walls of the right atrium (RA) and right ventricle (RV) (Shiotani et al. 2005). The causes of such CVG are venous catheterization that permits possible air inflow and pneumatization of dissolved gas in the blood as a result of chest compression (Shiotani et al. 2005). However, to our knowledge, there has been no paper which describes myocardial intravascular gas of the left ventricle (LV) as a CPR-related change. Herein, we report a sudden death case involving CPR and in which PMCT images showed gas of the LV immediately after death.

## Case report

An 87-year-old man suddenly mis-swallowed food, impairing respiration, which caused him to collapse and lose consciousness. When emergency technicians arrived at the site, he was in a state of cardiopulmonary arrest (CPA). CPR was performed for 40 min by emergency technicians during transport to our emergency room (ER) and by emergency medical physicians for an additional 30 min in our ER. CPR was performed by continuous external chest compression, artificial respiration with bag-valve mask ventilation following endotracheal intubation, and peripheral intravenous catheterization following administration of epinephrine at 1 mg. However, the CPR was ineffective and death was confirmed.

After investigation of the location where the man was found in CPA, and subsequent cadaveric examination, the police rejected the cause of death due to traumatic accident or criminal reason. As the cause of death re-mained to be undetermined and the family did not want an autopsy done, PMCT was performed immediately after the confirmation of death using a clinical scanner in the Radiology Department of our institution with the prior approval of the institutional review board. The corpse exhibited no signs of putrefaction at the time of the scan.

PMCT was performed with a 64-channel multidetector-row CT scanner (Lightspeed VCT; GE Healthcare, Milwaukee, USA). The scan parameters for the thorax, abdomen, and pelvis were determined for helical scan mode with settings of auto mA (standard deviation value: 20), 120 kV, 1.0 second/rotation, 0.625 mm collimation, pitch 1.375, and contiguous 1.3-mm sections. The scan parameters for the heart were determined for conventional scan mode with settings of auto mA, 120 kV, 2.0 seconds/rotation, 0.625 mm collimation, and contiguous 1.3-mm sections. We obtained image reformations of the contiguous 1.3 mm sections in short-axis and horizontal long-axis plane from 0.625 mm axial reconstructions. All images were observed on a 21-inch monochrome monitor with a resolution of 1,600 × 1,200 pixels at appropriate window settings for each region. A board-certified diagnostic radiologist with 21 years of experience and a subspecialty in forensic radiology judged CVG to be present when air density, which is lower than that of the lung, was observed in the vessels at the lung window setting (window level/width = -600/1600).

PMCT of the thorax showed airless bilateral bronchi filled with fluid. Based on a comprehensive understanding of the patient’s clinical course and PMCT findings, cause of death was diagnosed mis-swallowing.

**Figure 1:**
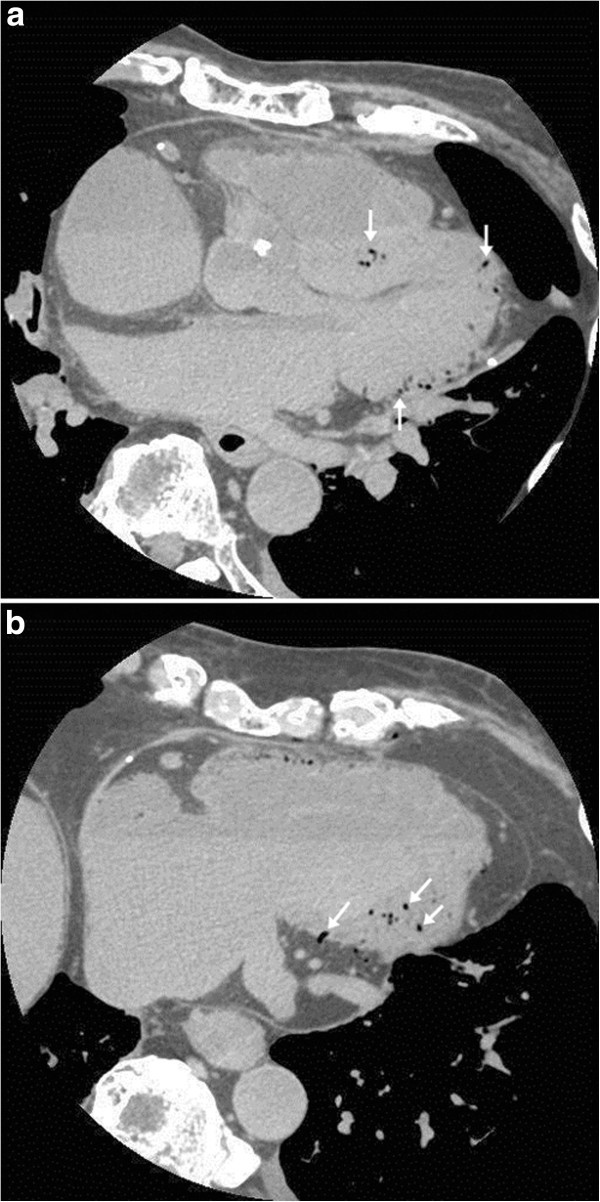
**Myocardial intravascular gas of the left ventricle on postmortem CT of the heart. a**. Axial image at the level of aortic root shows intravascular gas in the ventricular septum, apex, and posterior wall *(arrows)*. **b**. Axial image at the level of inferior wall shows intravascular gas in the inferior wall *(arrows)*.

Multiple intravascular gases were shown by PMCT in the brain, bilateral jugular veins, bilateral brachiocephalic veins, RA, RV, LV, and the liver. CVG in the LV was located in the myocardium and appeared as a spotty, linear, or branch-shaped, suggesting the presence of myocardial intravascular gas (Figure 1). The exact location of these gases could not be differentiated as either arterial gas, venous gas, or both. Vessels in the epicardial fat connected with coronary sinus contained spotty gas, suggesting the gas was intravenous.

## Discussion

To evaluate intravascular gas as a CPR-related change, PMCT examination needs to be done as soon as possible after death, to avoid effects from putrefaction. The patient in this study underwent PMCT immediately after confirmation of death, and thus the effects from putrefaction are considered minimal.

One of the causes of CVG on early non-traumatic PMCT is pneumatization of dissolved gas in the blood as a result of chest compression (Shiotani et al. 2005). This physiological phenomenon is called tribonucleation (= exercise-induced cavitation) and can generate gas nuclei (Roston & Haines 1947; Ikels 1970). The veins are more likely to collapse and expand in response to CPR than the arteries, and gas nuclei tend to be produced (Ikels 1970). Therefore, myocardial intravascular gas of the LV is considered to have occurred intravenously. However, tribonucleation alone cannot explain why CVG is seen in the RA, RV, and myocardial vein of the LV, but not in the LA. The thoracic pump theory suggests that chest compression produces a rise in the intrathoracic pressure that is transmitted equally to all intrathoracic vascular structures (Criley et al. 1976; Radikoff et al. 1980; Babbs 1980).

The common factor among the RA, RV, and the myocardial vein of the LV is that these include venous blood and the partial pressure of carbon dioxide (*P*CO_2_) is high. Gas nuclei grow to become visible, and stable gas bubbles depend on the dissolved CO_2_ gas concentration (Wilbur et al. 2010; Lin et al. 2000). Partial venous pressure of CO_2_ (*P*vCO_2_) elevates during CPR and its causes are as follows (Suwa 1992; Halmagyi et al. 1970; Suwa et al. 1969; Chazan et al. 1968; Weil et al. 1986; Gudipati et al. 1990; Lindner et al. 1991): 1) increased arterial-venous difference of *P*CO_2_ due to low cardiac output, 2) increased CO_2_ production due to anaerobic metabolism progression, and 3) decreased CO_2_ transport by the blood. Normal partial arterial pressure (*P*a) and partial venous pressure (*P*v) are as follows (Hills 1985; Liew et al. 1993): *P*aH_2_O = *P*vH_2_O = 47 mmHg, *P*aN_2_ = *P*vN_2_ = 573 mmHg, *P*aCO_2_ = 40 mmHg, *P*vCO_2_ = 46 mmHg, *P*aO_2_ = 88 mmHg, *P*vO_2_ = 40 mmHg, *P*a inherent unsaturation (IU) = 12 mmHg, and *P*vIU = 54 mmHg. During CPR, *P*H_2_O (47 mmHg) and *P*N_2_ (573 mmHg) are stable, but the *P*O_2_ and *P*CO_2_ change. In cases where the outcome of CPR is not successful, *P*vCO_2_ markedly increases, and this additional CO_2_ may be sufficient to exceed the IU, and the total pressure in the venous blood exceeds atmospheric pressure (760 mmHg); consequently the venous blood is supersaturated and releases its excess solute CO_2_ as gas bubbles (Hills 1977; Rasmussen et al. 1999). CVG in the RA, RV, and myocardial vein of the LV are changes related to CPR, and may be a sign of death.

Our study has some limitations. First, the rate of occurrence is still unknown regarding the appearance of myocardial intravascular gas in the LV on non-traumatic PMCT performed immediately after certification of death as a CPR-related change. Although this phenomenon is anticipated to occur as frequently as CVG in the RA and RV, the occurrence may depend on the strength and duration of chest compression. Second, PMCT findings could not morphologically differentiate whether the myocardial intravascular gas of the LV was in the artery or in the vein. However, tribonucleation and supersaturation of CO_2_, which are the two predominant theories of gas nuclei and bubbles origin (Wilbur et al. 2010; Lin et al. 2000), suggest that the gases were in the vein. Additionally, the presence of intravascular gas in the venula of pericardial fat may be indirect evidence. Third, it was not clear whether or not the major component of the myocardial intravascular gas of the LV was CO_2_. As collection and analysis of the gas during autopsy is difficult, it is not possible to determine the gas component. These limitations may be overcome by further experimental and clinical study. Meanwhile, the promising results presented here may serve to guide interpretation of PMCT following CPR.

## Abbreviations

RA: Right atrium
RV: Right ventricle
LA: Left atrium
LV: Left ventricle
P: Partial pressure
*P*a: Arterial partial pressure
*P*v: Venous partial pressure
CO_2_: Carbon dioxide
O_2_: Oxygen
N2: Nitrogen
H_2_O: Water
IU: Inherent unsaturation.
